# Microsatellite Marker Discovery in the Stingless Bee Uruçu-Amarela (*Melipona rufiventris* Group, Hymenoptera, Meliponini) for Population Genetic Analysis

**DOI:** 10.3390/insects10120450

**Published:** 2019-12-13

**Authors:** Aline B. Negreiros, Geice R. Silva, Francisca A. S. Oliveira, Helder C. Resende, Tânia M. Fernandes-Salomão, Rodrigo Maggioni, Fabia M. Pereira, Bruno A. Souza, Maria T. R. Lopes, Fábio M. Diniz

**Affiliations:** 1 Northeast Biotechnology Network—RENORBIO/Animal Science Program—PPGCA, Universidade Federal do Piauí, Teresina PI 64049-550, Brazil; aline.negreiros@ifpi.edu.br (A.B.N.); geiceamb_bio@yahoo.com.br (G.R.S.); 2 Institute of Marine Sciences, LABOMAR, Universidade Federal do Ceará, Fortaleza CE 60165-081, Brazil; andreasilvaoli@gmail.com (F.A.S.O.); maggioni@ufc.br (R.M.); 3 Instituto de Ciências Biológicas e da Saúde, Laboratório de Genética da Conservação, Laboratório de Genética da Conservação, campus UFV Florestal, Universidade Federal de Viçosa, IBF, Florestal MG 35690-000, Brazil; helder.resende@ufv.br (H.C.R.); fernands@ufv.br (T.M.F.-S.); 4 Laboratory of Molecular Biology & Biotechnology, EMBRAPA Meio-Norte, CP: 01, Teresina PI 64.006-220, Brazil; fabia.pereira@embrapa.br (F.M.P.); bruno.souza@embrapa.br (B.A.S.); maria-teresa.lopes@embrapa.br (M.T.R.L.); 5 EMBRAPA Caprinos e Ovinos, Sobral CE 62010-970, Brazil

**Keywords:** conservation, genetic differentiation, Illumina platform, molecular markers, NGS technology, population genetics

## Abstract

The species *Melipona rufiventris* Lepeletier, 1836 is a Brazilian native stingless bee that is part of a species complex known as the ‘*rufiventris* group’, making it difficult to distinguish between the different species. Populations in this group are facing a severe decline, leading to the risk of local extinction, and therefore, their conservation should be treated as a major concern. This study describes the first set of tri- and tetranucleotide microsatellite markers, using next-generation sequencing technology for use in the identification of genetic diversity and population structure in the ‘*rufiventris* group’. A total of 16 microsatellite *loci* displayed polymorphism. Analysis of the whole data set (n = 50) detected 63 alleles in all *loci*, ranging from 2 to 7 with a mean of 3.9 alleles/locus. A genetic diversity analysis revealed high values for population differentiation estimates (*F*_ST_ = 0.252, *R*_ST_ = 0.317, and *D*_EST_ = 0.284) between the Atlantic Forest, Cerrado, and Caatinga biomes. An additional evidence for genetic divergence among populations was also found in the ’*rufiventris* group’; these should be treated as separate conservation units or even as separate species. These microsatellite markers have demonstrated a strong potential for assessing population discrimination in this threatened stingless bee group.

## 1. Introduction

Bees are considered the main pollinators in natural and agricultural environments. This ecosystem service is essential to sustaining species diversity and production of crops. Meliponines belong to this group of eusocial insects and are characterized by atrophied stingers in both workers and queens, and are found in most tropical or subtropical regions of the world [1].

The species *Melipona rufiventris* Lepeletier, 1836 is a native stingless bee distributed mainly in the Atlantic Forest, Caatinga, and Cerrado biomes in Brazil [1,2]. It is a polytypic species that is part of a group known as the ‘*rufiventris* group’ because of their similar morphology, which makes it difficult to distinguish between the different species [3,4,5]. Additional biological data are necessary for a better identification of the taxonomic status of these species. Most of the literature suggests that *M. rufiventris* is a complex of two or three species with a distinct distribution [2,6,7]. This native stingless bee group has a limited flight capacity compared to *Apis mellifera* [8,9], and when associated with geographic boundaries, it can entail low dispersal rates among populations. Moreover, queen mating in *M. rufiventris* occurs with a single male, and as a result, considerably low intracolonial genetic variability can be generated [10].

Some of the Brazilian endemic stingless bees are on the official national list of fauna species threatened with extinction [11]. The species *Melipona rufiventris* is one of these bees that are becoming rare, and are listed as Endangered (EN), due to the adverse effects of deforestation, habitat loss, and predatory collection of honey [5,12]. Therefore, its conservation should be treated as a major concern, aiming at the adequate management of this important genetic resource and the implementation of efficient conservation strategies, securing pollination services in both commercial agriculture and natural ecosystems.

Microsatellite markers, or simple sequence repeats (SSR), have emerged as one of the most popular and effective tools for determining the genetic divergence among populations and the estimation of population structure and genetic diversity in different taxa [12,13,14], which is essential for the development of efficient conservation strategies. The use of microsatellites as an advanced tool is of fundamental importance in the estimation of possible disturbances caused in natural bee populations. Microsatellites allow the assessment of not only the structural situation of genetic composition but also of the reproductive behavior, social structure, and dispersal in endangered bee species [15].

There are very few species-specific microsatellite markers for *M. rufiventris* (eight markers; [10]), and most are dinucleotides presenting low repeat numbers (≤8), which might affect the level of polymorphism detected by these available markers. The polymorphism in a microsatellite locus depends on the number of repeats it contains, and the level of polymorphism increases with the number of repeats [15,16]. Moreover, long core motifs (i.e., tri- and tetranucleotides or longer), enable a better separation of alleles, compared to dinucleotide repeats, which more frequently produce stutter peaks, making it difficult to correctly interpret electropherograms of microsatellite genotyping [17].

Cross-species amplification of microsatellite *loci* from primers developed from other species in the same genus (e.g., *Melipona bicolor* [18], *M. seminigra merrillae* [19], *M. interrupta manaosensis* [20], *M. mondury* [21], *M. subnitida* [22] and *M. fasciculata* [23]) might occur mostly due to the presence of conserved flanking sequences across closely related taxa. However, the use of heterologous microsatellite primers in a non-source species, where nonamplifying (null) alleles are expected, could substantially reduce genetic variability, and consequently, the resolving power of the marker [24]. Thus, it is expected that species-specific tri- and tetranucleotide markers will provide a better dataset than previously identified markers [10] in improving our understanding of the population structure and diversity of the stingless bee *Melipona rufiventris*.

Next generation sequencing has been recently used for the isolation and development of microsatellite loci in the *Melipona* species [22,23]. The technology has the advantage over traditional methods used for the discovery of SSR markers, as it generates large amounts of sequencing data and consequently large numbers of markers in a simpler and rapid approach, avoiding the construction of microsatellite-enriched DNA libraries, which is a time-consuming and laborious procedure [25].

Therefore, this study describes the first set of tri- and tetranucleotide microsatellite markers, using low coverage whole genome sequencing data from the Illumina platform, and their application to assess genetic diversity and population structure of the existing forms in the ‘*Melipona rufiventris* group’ in three Brazilian biomes.

## 2. Materials and Methods

### 2.1. Sampling and DNA Extraction

**Figure 1:**
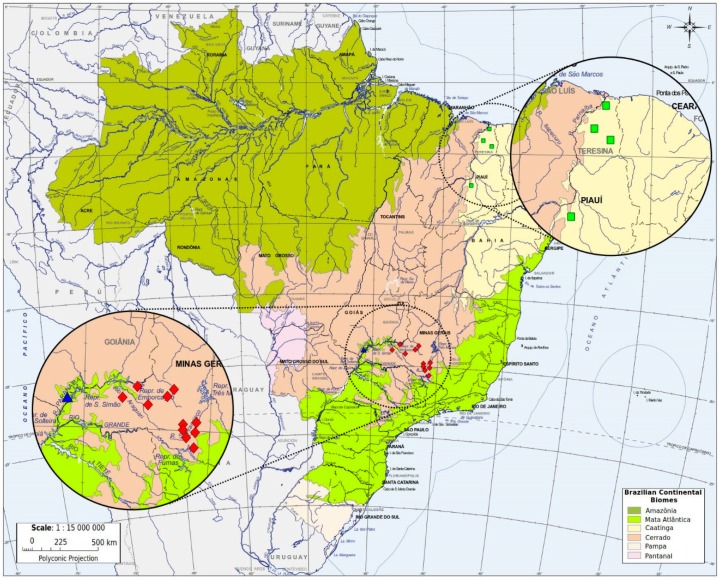
Sampling locations of the ‘*Melipona rufiventris* group’ in Brazilian continental biomes. Atlantic Forest (▲), Caatinga (■), and Cerrado (υ). The map of Brazil used for the elaboration of Figure 1, and the shapefile to generate it, were extracted from the database of public domain of the Brazilian Institute of Geography and Statistics-IBGE (http://mapas.ibge.gov.br/bases-e-referenciais.html), and was further modified using Inkscape 0.91 (https://inkscape.org/en/).

A total of 50 worker bees, one from each colony, were randomly collected in the states of Minas Gerais (n = 20), Goiás (n = 15), and Piauí (n = 15), Brazil (Figure 1, Table 1). Total genomic DNA was extracted from the thorax of each bee using the ExtractME Genomic DNA Kit (*DNA*-Gdansk), following the manufacturer’s protocol for animal tissue. DNA samples were electrophoresed on 1.0% agarose gel to test for overall quantity and quality of the DNA yield.

### 2.2. Library Preparation and High-Throughput Sequencing

A single individual DNA sample of approximately 1 µg was used to prepare the genomic library for sequencing, following the standard protocol of the Illumina Nextera XT Library Preparation kit (Illumina Inc., San Diego, CA, USA). The DNA library was sequenced using a MiSeq Benchtop Sequencer (Illumina Inc., San Diego, CA, USA), targeting 500-bp fragments with 2 × 250-bp reads in a paired-end sequencing configuration. The paired-end Illumina reads were first combined to produce contigs with CLC Genomics Workbench 7.0.4 (Qiagen, Redwood City, CA, USA).

### 2.3. SSR Mining and Primer Design

Contigs were added directly into Msatcommander 0.8.2 [26] for detection of possible microsatellite loci with at least six repeats for tri- and tetranucleotides. Primers, forward and reverse, were designed for each short tandem repeat sequence at their flanking regions. Long mononucleotide repeats [(A)n, (G)n, (C)n, (T)n, n > 5] between primer annealing locations were avoided for marker development. Primer design was performed with the web-based Primer3 program (http://bioinfo.ut.ee/primer3-0.4.0/).

### 2.4. PCR Amplification and Validation of Selected SSRs

Genomic DNA from the 50 individuals described above (Table 1) were used to validate all designed primer pairs using polymerase chain reactions (PCRs), and to obtain baseline allele frequency information. Reactions were performed in a 10-µL total volume containing at least 20 ng of genomic DNA, with 1.0 × buffer, 2 to 3 mM MgCl_2_, 1.0 mM dNTP mix, 0.25 mM of each primer and 0.75 units of *Taq* DNA polymerase (Thermo Scientific Inc, Waltham, MA, USA). All reactions were run on a Veriti 96-well Thermal Cycler (Applied Biosystems, Waltham, MA, USA) using the PCR temperature profile indicated in Table 2. Amplicons were screened by silver nitrate detection on denatured 6% polyacrylamide gels.

### 2.5. Data Analysis

The software Micro-Checker 2.2.3 [27] was used to detect null alleles and scoring problems in the genotyped data. Observed heterozygosity (*H*_O_), expected heterozygosity (*H*_E_), and allele richness (*A*_R_) were calculated using the Fstat v2.9.3.2 software [28]. The polymorphic information content (PIC) was determined using Cervus ver. 3.0 [29]. Deviations from the Hardy–Weinberg equilibrium (HWE) and tests for linkage disequilibrium were conducted using the Genepop software [30]. A Bayesian grouping admixture model with no population assumed a priori, implemented in the software Structure 2.3.4 [31], was used to identify genetically homogeneous groups within the genotyped data. The estimate of the best K, number of groups that best fit the data, was calculated based on 5 replications for each K (from 1 to 11) using Structure Harvester v.0.6.92 [32]. The program was set up for 1,000,000 Markov chain Monte Carlo repetitions, following a burn-in-period of 500,000 iterations. Population structure was also analyzed using principal coordinate analysis (PCoA), *R*_ST_, a measure of genetic differentiation analogous to *F_ST_*, and *D*_EST_ estimator of actual differentiation, as implemented in GenAlEx v.6.5 [33].

## 3. Results and Discussion

### 3.1. Sequence Assembly and SSR Mining

Illumina MiSeq sequencing resulted in 54,555,929 reads, which were assembled into a total of 137,313 contig sequences. Minimum and maximum contigs were 200 and 13,505 bases, respectively, with an average size of 397 bases. The Msatcommander 0.8.2 program identified 9745 (7.1%) contigs with microsatellite loci consisting of di- to hexa-nucleotide SSRs with at least six repetitions.

For ease of imaging and scoring, only tri- and tetranucleotides were examined. From these potential microsatellite markers, 25 loci were randomly selected for primer designing and validation in *Melipona rufiventris*. All microsatellite sequences isolated and validated in this study were deposited in the NCBI in the GenBank database under accession numbers MK133898–MK133922.

### 3.2. SSR Validation

Among all 25 designed primer pairs, 16 loci (64%) were amplified successfully, producing consistent and specific PCR bands of expected size. Possible assembling errors or mutations in both primer-annealing sites at each locus could have resulted in the failed amplifications.

Analysis of the whole data set (n = 50) revealed 63 alleles in all 16 loci, ranging from 2 (*Mruf 6*, *13*, *19*, *20*, *21*, *22*) to 7 (*Mruf 9*) with a mean of 3.9 alleles/locus (Table 2). This result corroborates previous findings on microsatellite development in closely related species, such as *Melipona seminigra merrillae* [19] (*N*_A_ = 3.7), *Melipona interrupta manaosensis* [20] (*N*_A_ = 2.8), *Melipona mondury* [21] (*N*_A_ = 3.0), *Melipona yucatanica* [13] (*N*_A_ = 2.6 to 3.6), and *Melipona fasciculata* [23] (*N*_A_ = 3.9). Furthermore, these novel microsatellite loci were more polymorphic than the SSR markers developed from an earlier study by traditional cloning methods on the same species (*N*_A_ = 2.6) [10].

All loci were observed to segregate independent of each other, showing no evidence of linkage disequilibrium (*p* > 0.05). The polymorphism of microsatellite loci was also separately evaluated in terms of allelic richness (*A*_R_), heterozygosities (*H*), and the polymorphic information content (PIC) in the samples collected from the three distinct biomes (Table 3). Mean *A*_R_, observed and expected heterozygosities (*H*_O_ and *H*_E_), and PIC were low to moderate, respectively, 2.4, 0.514/0.425, 0.345 for Minas Gerais in the Cerrado biome, 2.7, 0.547/0.479, 0.400 for Goiás in the Atlantic Forest, and 2.7, 0.563/0.502, 0.412 for Piauí in the Caatinga. Despite the fact that the low levels of genetic diversity might occur in social Hymenoptera compared to other insects [34], recent population reduction might also have contributed to this alarming scenario, reducing the genetic variability in these populations of the ’*rufiventris* group’ [5,35]. Heterozygosity estimates were of similar magnitude when compared to those found for *Melipona fasciculata* [23], but were a little lower for *M. subnitida* [22].

From the 48 combinations (16 loci and 3 populations) of the overall samples, 10 significant deviations from HWE after sequential Bonferroni correction (*p <* 0.0031) were detected, mostly towards an excess of heterozygotes. This could be attributed to a reduction in population size, probably associated with extensive deforestation for new land use and urban expansion, which leads to a disproportionate loss of rare alleles, such that predicting heterozygosity from allele numbers alone creates an underestimated value [36,37]. Additionally, null alleles, inbreeding or selection for or against a certain allele could also have played a part in the departures of HWE [24]. Evidence for null alleles was found only for *Mruf1* in Goiás and Piauí, at estimated frequencies of 0.216 and 0.218, respectively, and for loci *Mruf9* (0.234) and *Mruf18* (0.261) in Minas Gerais.

### 3.3. Genetic Divergence Among Populations in the ’Rufiventris Group’’

In our study, *F*_ST_ (0.252), *R*_ST_ (0.317), and D_EST_ (0.284) estimates obtained from the microsatellite loci suggest the existence of genetic differentiation among populations/colonies in the ’*rufiventris* group’ (Table 3). Even though these estimates are obtained through different computational methods, they were of a broadly similar magnitude, indicating significant genetic differences among the samples collected in the three contrasting biomes. Admittedly, a more extensive sampling effort over a wider spatial range in the Atlantic Forest was needed. Values of *F*_ST_ > 0.25 indicate very high genetic differentiation, which can be caused by natural selection or of limited gene flow between populations [38]. Our results are consonant with earlier observations in *Melipona rufiventris* [12] (*F*_ST_ = 0.250) and in *M. beecheii* [39] (*F*_ST_ = 0.280). Such high differentiation might reflect partial isolation with reduced gene flow between the colonies located in different biomes, resulting in high inbreeding within localities [40]. A low migration rate between populations of *M. rufiventris*, 0.055 bees per generation, was detected from microsatellite data collected in Minas Gerais [12].

**Figure 2:**
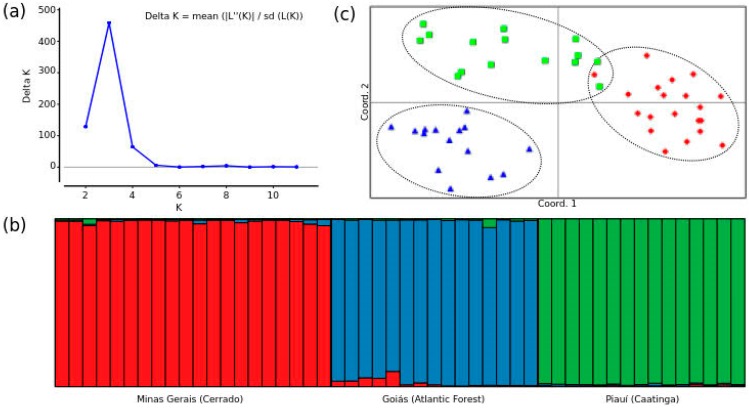
(**a**) Determination of the best number of genetic clusters from STRUCTURE analysis for microsatellite loci in ‘*Melipona rufiventris* group’ colonies; K is the number of clusters. (**b**) Bar plot from inferred population structure using the Bayesian grouping admixture model-based program STRUCTURE (K = 2). (**c**) Scatter-plot of the principal coordinate analysis (PCoA) using SSR loci. υ Minas Gerais (Cerrado); ▲ Goiás (Atlantic Forest); and ■ Piauí (Caatinga).

The admixture model-based clustering (Structure analysis) recognized three distinct genetic populations. The ad hoc statistic ∆K, used to infer the true number of clusters (K) that capture the major structure of the dataset, revealed the best K at the second level of sub-population separation (K = 3), with a strong signal (∆K > 400), as seen in Figure 2a. These clusters represent a biologically meaningful level of organization within the colonies surveyed in this study, considering three distinct biomes (Figure 2b). High population genetic structure suggests low levels of gene flow among the colonies from distinct biomes that might not be sufficient to counterbalance genetic drift [41].

Principal coordinate analysis (PCoA) of codominant genotypic distance was conducted to obtain further insights into the relationships among populations of the ‘*rufiventris* group’. The analysis corroborated the population assignments inferred by Structure and the pairwise *F_ST_* calculations (Figure 2c). Colonies from Goiás, in the Atlantic Forest biome, diverge along both PCoA axes, whereas colonies from Minas Gerais and Piauí, even though clearly distinct, showed very little overlap in the distribution of individuals. All colonies, however, were clearly clustered into three different groups forming separate genetic clusters and, thus, suggesting a high genetic differentiation. In fact, individuals from colonies occurring in the Atlantic Forest showed morphological differences that are not described for bees in other regions, and according to Melo [2] these two forms should be treated as two different species. The variety found in the Cerrado biome (Minas Gerais) should be called *Melipona rufiventris* Lepeletier 1836, while the form found in the Atlantic Forest (Goiás) should be called *M. mondury* Smith 1863 [2,6]. Our study also suggests that a third form exists in the Caatinga (Piauí), in agreement with results from previous research [7] that indicates a new species in the ‘*rufiventris* group’, with a distribution that goes from the northwest of Minas Gerais to Maranhão, including the state of Piauí.

## 4. Conclusions

In summary, our findings showed that the set of molecular markers described here—which are the first microsatellite loci for the ‘*rufiventris* group’—using NGS technology, have a strong potential for population-level genetic studies, and consequently, will add valuable information to aid in the conservation and management of the species. The information obtained by this set of markers could be used in conjunction with ecological data to genetically monitor the ‘*rufiventris* group’, in order to help detect populations at risk of decline and improve population viability for ensuring appropriate conservation and management decisions.

Further support is also given to the presence of high degree of genetic differentiation in the ‘*rufiventris* group’, representing colonies from three distinct biomes, and that these should be treated as separate conservation units, each managed accordingly. However, further investigation should be conducted to confirm the extent of this genetic delimitation.

## Figures and Tables

**Table 1:** Location and number of *Melipona rufiventris* (‘*rufiventris* group’) colonies and worker bees sampled within three Brazilian continental biomes.

Localities	State	Biome	Latitude/Longitude	Number of Colonies (Worker Bees)
Guimarânia	Minas Gerais	Cerrado	−18°50′/−46°47′	4 (4)
Araguari	Minas Gerais	Cerrado	−18°38′/−48°11′	3 (3)
Patos de Minas	Minas Gerais	Cerrado	−18°34′/−46°31′	1 (1)
Uberlândia	Minas Gerais	Cerrado	−18°55′/−48°16′	1 (1)
Águas do Paraíso	Minas Gerais	Cerrado	−20°19′/−45°27′	1 (1)
Iguatama	Minas Gerais	Cerrado	−20°10′/−45°42′	2 (2)
Tapiraí	Minas Gerais	Cerrado	−19°53′/−46°01′	1 (1)
Bambuí	Minas Gerais	Cerrado	−20°00′/−45°58′	1 (1)
Arcos	Minas Gerais	Cerrado	−20°16′/−45°32′	4 (4)
Lagoa da Prata	Minas Gerais	Cerrado	−20°01′/−45°32′	1 (1)
Córrego Danta	Minas Gerais	Cerrado	−19°49′/−45°54′	1 (1)
Caçu	Goiás	Atlantic Forest	−18°33′/−51°07′	5 (15)
Murici dos Portelas	Piauí	Caatinga	−03°19′/−42°05′	3 (4)
Campo Maior	Piauí	Caatinga	−04°49′/−42°10′	2 (5)
Castelo do Piauí	Piauí	Caatinga	−05°19′/−41°33′	1 (3)
Guadalupe	Piauí	Caatinga	−06°47′/−43°34′	1 (3)
Total	-	-	-	32 (50)

**Table 2:** Characteristics and amplification of 16 polymorphic microsatellite markers developed for *Melipona rufiventris* (N = 50).

Locus	Primer Sequence (5′–3′)	Motifs Repeats	PCR Profile	Ta (°C)	Size Range (bp)	*N*_A_	GenBank Accession No.
*Mruf* 1	F: CAGTCGCCCAAGTAAATACG R: CTTATGAAACGAACCACAAGCC	(AGCC)_8_	PCR_STD1_	54	142–166	6	MK133898
*Mruf* 4	F: GTTACGTTGGCAGGAGAGC R: AACTTGATTATTAGCGCGTGA	(TGCT)_8_	PCR_STD1_	52	146−166	6	MK133901
*Mruf* 5	F: AGTGAAATCCGAGAGTGGGTT R: TCTCCACCGTCTTTTGTTTCTT	(AGAA)_9_	PCR_STD1_	51	154−170	4	MK133902
*Mruf* 6	F: GTGCCTCGTTACCACCTTCTC R: TTAAAAGTGCGACGGGGA	(CT)_9_N_13_(GTCT)_6_	PCR_STD1_	54	106−110	2	MK133903
*Mruf* 8	F: CATCGTCCTCCCGTGAATATAG R: TGCTTTTCCTTCCACGACC	(GCTG)_10_	PCR_STD1_	50	102−122	5	MK133905
*Mruf* 9	F: TATACTTACGAGAGCGCACGAG R: TATTTTCTACGGTCCCACTTCG	(AACG)_9_	PCR_STD1_	55	118−140	**7**	MK133906
*Mruf* 11	F: TGTGACGTTTTGGACGTAATTC R: CGCTTCCTTTGATCTCTCGAT	(TTTA)_13_	PCR_STD2_	48	112−124	4	MK133908
*Mruf* 13	F: GCTAGGGGACCTTCTTCTTCTT R: GTGATAAGGCGGAGTGTAATC	(TTC)_12_	PCR_STD1_	50	100−106	2	MK133910
*Mruf* 15	F: AAGTGGGGAGCTAATAAGGGAG R: TGCAGAGGAGCAGTACAGAGAG	(TTC)_12_	PCR_STD3_	51	157−172	4	MK133912
*Mruf* 17	F: GTCGAGGACGACTACACAACAA R: CTCACCGCACACAGGGTT	(ACG)_15_	PCR_STD1_	52	161−173	3	MK133914
*Mruf* 18	F: AAGCGGACAAGCAGATCACT R: ACTGTATGTCGTTCCTCGTCCT	(AAT)_9_	PCR_STD1_	52	108−126	6	MK133915
*Mruf* 19	F: CACTGTCTTGTATTTAGACGCAATC R: GGTCGGGGACTTTAGTGTTTTA	(TTG)_14_	PCR_STD1_	55	125−134	2	MK133916
*Mruf* 20	F: CGGGTAGTATTAAGGGAATTGA R: TGTGTCAGGAAGAAAAGCAA	(ATT)_9_	PCR_STD1_	54	184−193	**2**	MK133917
*Mruf* 21	F: CTACCGAGAGTAGCGACGACAT R: TCAGTTCTCAATGTTGCAGGC	(ACG)_11_	PCR_STD1_	54	150−162	2	MK133918
*Mruf* 22	F: CGACTTCGCGTGGTGCTAC R: AGAGGTTTCGGCGGCTTC	(ACG)_9_	PCR_STD1_	54	125−131	2	MK133919
*Mruf* 25	F: AACAAGAGCAAAGTAACGACGA R: GAAGGAACAAGTCGAAACCAAC	(ACG)_7_N_11_(GAA)_13_	PCR_STD3_	51	137–155	6	MK133922

Ta: Annealing temperature; *N*_A_: Number of alleles; PCRSTD1: (94 °C–5 min; 30 cycles (94 °C–40 s; Ta–30 s; 72 °C–40 s); 72 °C–7 min); PCRSTD2: (94 °C–5 min; 45 cycles (94 °C–40 s; Ta–30 s; 72 °C–40 s); 72 °C–7 min); PCRSTD3: (94 °C–5 min; 40 cycles (94 °C–40 s; Ta–30 s; 72 °C–40 s); 72 °C–7 min).

**Table 3:** Variability of 16 microsatellite *loci* and genetic diversity estimates in the ‘*Melipona rufiventris* group’.

*Locus*	Minas Gerais (n = 20)					Goiás (n = 15)					Piauí (n = 15)					*F*_ST_	*R*_ST_	*D*_EST_
	*A*_R_	*H*_O_/*H*_E_	PIC	pHWE	Null	*A*_R_	*H*_O_/*H*_E_	PIC	pHWE	Null	*A*_R_	*H*_O_/*H*_E_	PIC	pHWE	Null			
*Mruf* 1	1.0	0.000/0.000	0.000	-	0.000	3.5	0.286/0.664	0.569	0.001 *	0.216	4.6	0.357/0.762	0.689	0.001 *	0.218	0.288 ^¶^	0.279 ^¶^	0.333 ^¶^
*Mruf* 4	2.9	0.500/0.472	0.410	0.664	−0.027	3.6	0.786/0.643	0.562	0.473	−0.102	3.0	0.429/0.593	0.501	0.224	0.091	0.329 ^¶^	0.676 ^¶^	0.622 ^¶^
*Mruf* 5	2.0	0.077/0.212	0.183	0.118	0.106	3.0	0.615/0.625	0.532	0.025	−0.009	3.0	0.429/0.569	0.485	0.355	0.077	0.287 ^¶^	0.428 ^¶^	0.357 ^¶^
*Mruf* 6	2,0	0.917/0.518	0.373	0.014	−0.281	1.0	0.000/0.000	0.000	−	0.000	2.0	1.000/0.520	0.375	0.001 *	−0.333	0.270 ^¶^	0.270 ^¶^	0.229 ^¶^
*Mruf* 8	2,0	0.400/0.337	0.269	1.000	−0.061	2.6	0.385/0.335	0.290	1.000	−0.047	3.8	0.500/0.540	0.482	0.166	0.011	0.433 ^¶^	0.363 ^¶^	0.508 ^¶^
*Mruf* 9	3.9	0.294/0.709	0.630	0.000 *	0.234	4.8	0.800/0.766	0.701	0.351	−0.035	3.9	0.556/0.732	0.631	0.036	0.080	0.058	0.160	0.193 ^¶^
*Mruf* 11	4.0	1.000/0.779	0.696	0.010	−0.147	3.8	0.786/0.717	0.633	0.000 *	−0.056	4.0	0.667/0.752	0.657	0.050	0.025	0.030	−0.014	0.083
*Mruf* 13	2.0	0.933/0.515	0.374	0.002 *	−0.291	2.0	0.818/0.506	0.367	0.066	−0.226	2.0	1.000/0.524	0.375	0.003 *	−0.333	0.002	0.002	0.002
*Mruf* 15	3.1	0.714/0.521	0.433	0.430	−0.141	2.7	0.308/0.283	0.255	1.000	−0.028	2.8	0.400/0.416	0.347	0.223	−0.004	0.053	0.085	0.035 ^¶^
*Mruf* 17	1.0	0.000/0.000	0.000	-	0.000	1.0	0.000/0.000	0.000	-	0.000	2.0	0.462/0.492	0.361	1.000	0.008	0.850 ^¶^	0.771 ^¶^	0.847 ^¶^
*Mruf* 18	2.4	0.053/0.437	0.354	0.000 *	0.261	4.0	0.833/0.775	0.695	0.005	−0.052	2.7	0.917/0.562	0.432	0.013	−0.246	0.309 ^¶^	0.333 ^¶^	0.613 ^¶^
*Mruf* 19	2.0	0.850/0.501	0.369	0.002 *	−0.243	2.0	1.000/0.517	0.375	0.000 *	−0.333	2.0	1.000/0.517	0.375	0.000 *	−0.333	0.005	0.005	0.005
*Mruf* 20	2.0	0.500/0.386	0.305	0.526	−0.091	2.0	0.308/0.271	0.226	1.000	−0.038	1.0	0.000/0.000	0.000	−	0.000	0.106	0.106	0.033
*Mruf* 21	2.0	0.375/0.315	0.258	1.000	−0.054	2.0	0.636/0.455	0.340	0.481	−0.141	2.0	0.250/0.233	0.195	1.000	−0.026	0.019	0.019	0.012
*Mruf* 22	2.0	0.800/0.513	0.375	0.022	−0.200	2.0	0.692/0.471	0.350	0.208	−0.165	2.0	0.333/0.287	0.239	1.000	−0.044	0.112 ^¶^	0.112 ^¶^	0.083 ^¶^
*Mruf* 25	3.5	0.813/0.579	0.498	0.206	−0.162	3.0	0.500/0.633	0.511	0.322	0.059	3.0	0.700/0.532	0.442	0.503	−0.130	0.278 ^¶^	0.649 ^¶^	0.491 ^¶^
Mean	2.4	0.514/0.425	0.345	-		2.7	0.547/0.479	0.400	-		2.7	0.563/0.502	0.412	-		0.252 ^¶^	0.317 ^¶^	0.284 ^¶^

***A*_R_**: Allelic richness; ***H*_O_**: Observed heterozygosity; *H*_E_: Expected heterozygosity; PIC: Polymorphic Information Content; pHWE: probabilities of departure from Hardy–Weinberg equilibrium; Null: Null alleles frequency. * Locus that deviated significantly from HWE after Bonferroni correction (adjusted critical *p <* 0.0029). **^¶^** Significant (*p <* 0.05). Negative null-allele frequencies are a software artefact and can be interpreted as zero.

## Data Availability

The datasets generated or analyzed during the current study are available in the GenBank repository (accession numbers MK133898-MK133922) but restrictions apply to the availability of these data, so they are not publicly available until article publication.
